# Trichloroacetic Acid as a Topical Treatment for Actinic Cheilitis

**DOI:** 10.1002/oto2.70132

**Published:** 2025-05-23

**Authors:** Victoria Kuta, S. Mark Taylor

**Affiliations:** ^1^ Queen Elizabeth II Health Sciences Center, Division of Otolaryngology–Head & Neck Surgery Dalhousie University Halifax Canada

**Keywords:** actinic cheilitis, chemical peel, topical treatment, trichloroacetic acid

## Abstract

**Importance:**

Actinic cheilitis is a condition of the lower lip with the potential for malignant transformation. Although many topical treatment options exist, most involve prolonged application periods with expected adverse effects that limit compliance.

**Objective:**

Trichloroacetic acid is a widely used chemical peel that has been used for the treatment of precancerous skin lesions. This study aims to study the efficacy of 35% trichloroacetic acid as a topical treatment for actinic cheilitis.

**Study Design:**

Prospective cohort study of patients with actinic cheilitis presenting to our institution between September 2020 and December 2023. After treatment completion, patients were followed twice yearly for a minimum of 2 years.

**Setting:**

Tertiary care center.

**Methods:**

All patients with actinic cheilitis presenting to an otolaryngologist‐head and neck surgeon at our institution within the study time frame were eligible. Exclusion criteria include patients <18 years of age, patients who were pregnant, and patients with a biopsy‐proven malignancy of the lip. A topical 35% trichloroacetic acid peel was applied to the lower lip in the minor procedure clinic following bilateral mental nerve blocks. Patients were brought back 1 month later for follow‐up ± a repeat treatment. Photos were taken prior to treatment and 1 month following their final treatment. The severity of actinic cheilitis was graded using a proposed grading scale for actinic cheilitis, and the burden of the condition was assessed using Skindex‐16 Surveys. Visual analog scales were used to study adverse events. Patients were monitored for remission and recurrence.

**Results:**

A total of 11 patients were enrolled, with the majority requiring one treatment to achieve clinical remission. All patients who completed their full treatment course entered clinical remission following their trichloroacetic acid treatment, and there have been no cases of recurrence to date. The most common reported side effects were redness and swelling. Patients reported a significant improvement in their quality of life following treatment.

**Conclusion:**

This study suggests that a 35% trichloroacetic acid peel is a safe, well‐tolerated, and effective treatment option for patients presenting with actinic cheilitis. Further follow‐up is indicated to study the longevity of the achieved results.

**Trial Registration:**

This study is registered on Clinicaltrials.gov (NCT04744103). https://clinicaltrials.gov/study/NCT04744103?locStr=Halifax,%20NS,%20Canada&country=Canada&state=Nova%20Scotia&city=Halifax&cond=actinic%20cheilitis&rank=1.

Actinic cheilitis is a condition of the lower lip characterized by grayish‐white areas of discoloration and blunting of the demarcation between the cutaneous lip and the mucosa. It is associated with heavy or chronic sun exposure^1^ and is more commonly found in males.^2^ Approximately 10% to 30% of cases undergo malignant transformation to squamous cell carcinoma (SCC), and therefore, treatment is recommended at early presentation stages.^3^ Diagnosis can be clinical, histopathological, or both.

Although many studies have compared treatment options for actinic cheilitis, there is still significant debate over how to best manage this condition. The chosen treatment regimen must result in complete remission while also preventing recurrence and malignant transformation. Current treatment options are vast, including both nonsurgical and surgical options. Common topical therapies include 5‐fluorouracil, imiquimod, ingenol mebutate, and diclofenac. More recently, photodynamic therapy has been brought forward as a potential treatment modality. From a surgical perspective, a vermilionectomy is typically the procedure of choice.^4^

Previous studies have reported a higher remission rate with surgical treatment (92.8%) compared to nonsurgical treatments (65.9%). In addition, the recurrence rate is lower for surgical treatment (8.4%) versus nonsurgical treatments (19.2%).^5^ Although these results seem to favor surgical excision of actinic cheilitis, the procedure is not without risk. Patients are subjected to the risk of bleeding and infection, pain, scarring, and dysesthesia. Thus, topical therapies are still an attractive option for many patients.

Trichloroacetic acid (TCA) is a widely used chemical peel with a vast range of applications, both cosmetic and therapeutic. Topical application of TCA leads to a coagulation of skin protein and destruction of the epidermis and upper dermis layer, followed by regeneration of the dermis and epidermis with the production of new, healthy keratinocytes and new collagen deposition.^6^, ^7^

The peeling depth of TCA is dependent on the concentration used. Historically, chemical peels were completed using concentrations of 40% to 60% TCA; however, higher concentrations were found to carry an increased risk of scarring.^8^ Standard TCA applications now range between 30% and 50%, which results in a medium depth peel, penetrating full thickness epidermis and into papillary dermis.^8^ Depending on the desired penetration depth, this standard preparation can be further diluted. A superficial peel, for example, is typically achieved with TCA concentrations between 10% and 30%.^7^

From a therapeutic perspective, TCA is a popular topical treatment for treating fine rhytids, hyperpigmentation, photodamage, and premalignant changes, such as actinic keratoses.^9^, ^10^ Despite this, TCA is not commonly used for actinic changes on the lips. Here, we propose an expansion of the application of TCA. This study aims to study the efficacy of 35% TCA as a topical treatment for actinic cheilitis

## Methods

This was a prospective cohort study of patients with actinic cheilitis presenting to our institution. Institutional research ethics board approvals were obtained from the Nova Scotia Health Authority Research Ethics Board.

### Patient Selection

All patients with a clinical diagnosis of actinic cheilitis presenting to the Division of Otolaryngology–Head and Neck Surgery at our institution between September 2020 and December 2023 were eligible to participate. Patients were recruited during the initial consultation with their surgeon. Exclusion criteria included patients younger than 18 years, patients who were pregnant, and patients with a biopsy‐proven malignancy of the lip.

### Procedure

**Figure 1:**
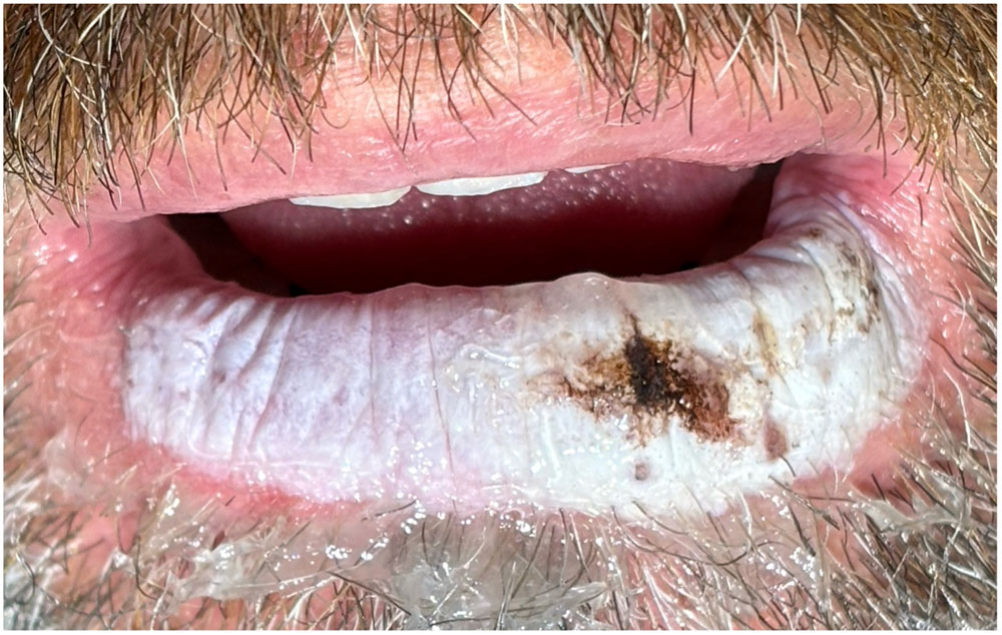
The lower lip during treatment. The white discolouration indicates activated trichloroacetic acid.

After their initial consultation, patients were booked for a minor procedure day. The procedure began with a transoral bilateral mental nerve block completed using a local injection of 1% lidocaine without epinephrine. The 35% TCA was then applied to the lip on a soaked Q‐Tip. This was rolled over the affected area until the tissue began to turn white, indicating that the TCA had activated on the tissues (Figure 1). The area was then neutralized with saline‐soaked gauze. Patients were instructed to apply Vaseline twice daily to the lower lip during the recovery period and were brought back in 1 month for follow‐up. Antivirals were not routinely administered before the peel. If a complete clinical remission was not achieved, a second peel was performed. This was completed at 1‐month intervals for a maximum of three treatments. After achieving clinical remission, patients were followed at 6‐month intervals to monitor for signs of recurrence. Patients were also counseled on the importance of sun protection for the lips.

### Variables of Interest

Photos were taken during the initial consultation and at the 1‐month follow‐up after the final treatment. A demographics questionnaire was administered. Patients completed a pretreatment and posttreatment Skindex‐16 Survey (Supplemental Appendix A1, available online), a validated measure of the effects of skin disease on quality of life. As there is no validated grading scale for actinic cheilitis, researchers graded the severity of the actinic cheilitis pretreatment and posttreatment using a proposed grading scale published by Poitevin et al (Supplemental Appendix A2, available online).^8^ Adverse outcomes were evaluated using patient reports on a 10‐point visual analog scale (VAS).

### Statistical Analysis

Descriptive variables were summarized using absolute (n) and relative (%) frequencies for categorical variables and mean and standard deviation (SD) for continuous variables.

All data were tested for normality using the Shapiro‐Wilk test. Paired *t* tests were used to evaluate the change in Skindex‐16 scores and actinic cheilitis grading scores pretreatment and posttreatment, with a mean and SD reported. A *P*‐value < .05 was considered statistically significant for these analyses.

Statistical analysis was performed using SPSS software version 29.0.2.0 (SPSS, Inc.).

## Results

A total of 11 patients met the inclusion criteria. The average age was 66.7 years (SD = 9.8), and the population was predominantly male (n = 7, 64%). All participants identified as Caucasian. The majority of participants categorized their lifetime sun exposure as average (n = 6, 55%), and more than half of the population recalled a facial sunburn in their history (n = 6, 55%). Baseline characteristics are found in Table 1.

**Table 1:** Demographic Characteristics and Exposure History of Study Cohort

Demographics	Value
Age, mean (range), y	66.7 (47‐82)
Sex, no. (%)	
Male	7 (64)
Female	4 (36)
Smoking, no. (%)	2 (18)
Immunocompromised, no. (%)	2 (18)
Sunscreen use, no. (%)	
Never	2 (18)
Rarely	2 (18)
Sometimes	4 (36)
Most times	1 (9)
Always	2 (18)
Lifetime sun exposure, no. (%)	
Average	6 (55)
Above average	4 (36)
High	1 (9)
Previous facial sunburn, no. (%)	6 (54)
Previous skin cancer, no. (%)	3 (27)

The average pretreatment actinic cheilitis grading was 3.2 (SD = 0.8), which improved to an average of 1.1 (SD = 0.3; *t* = 9.8, *P* = <.001) posttreatment. Our study did include one patient who was graded clinically as a two posttreatment. The clinical team recommended one additional treatment; however, the patient was satisfied with their result and chose not to pursue any further treatment.

A paired samples *t* test showed that the impact of actinic cheilitis on participants' quality of life significantly decreased from pretreatment (*M* = 27.8, SD = 21.3) to posttreatment (*M* = 6.9, SD = 12.9; *t* = 2.9, *P* = .014).

**Figure 2:**
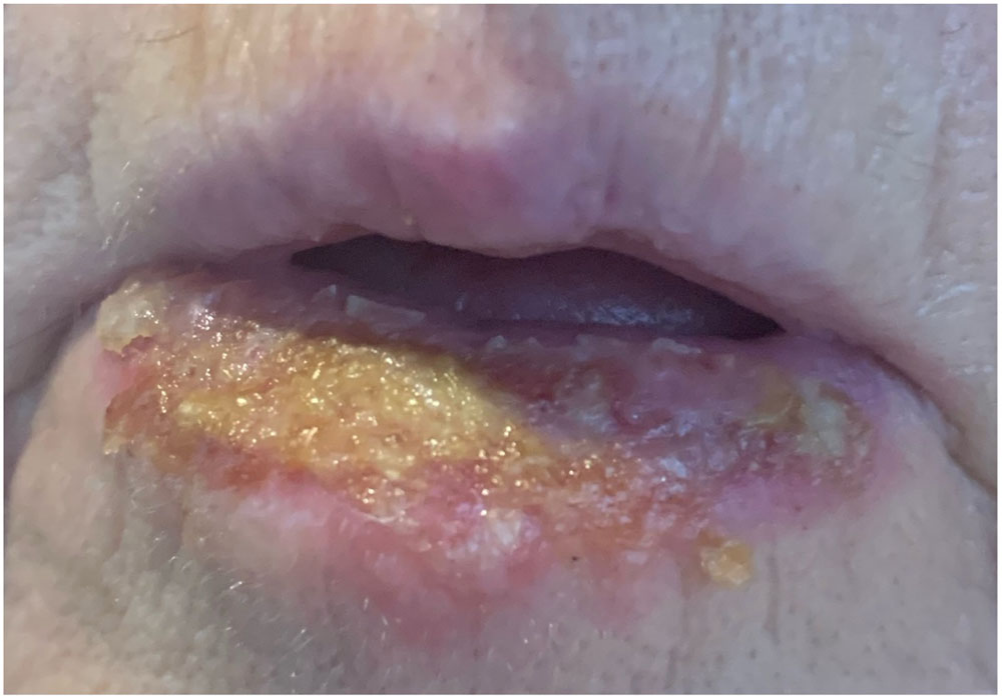
The treated lower lip during the acute healing phase.

The majority of patients saw resolution of their actinic cheilitis with one treatment (n = 8, 73%). The remainder were recommended for a second treatment. All patients who completed this achieved clinical remission (n = 2, 18%). The most common complication was swelling (VAS 4.3 [SD = 3.2]), followed by redness (VAS 3.5 [SD = 3.1]). The average healing time following a treatment was 2.6 weeks (SD = 1.1). The lower lip during the acute healing phase can be seen in Figure 2.

**Figure 3:**
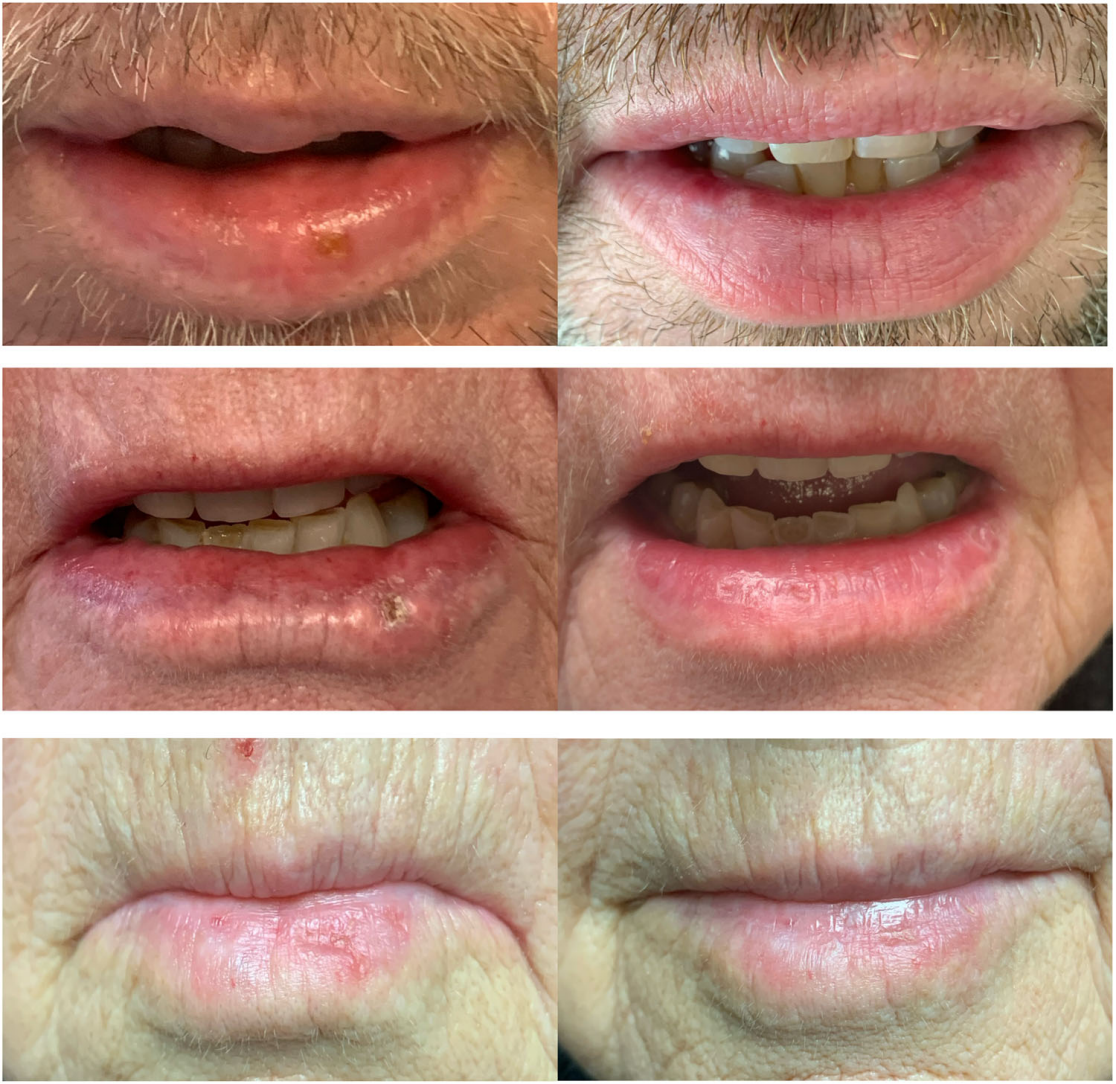
Before and after trichloroacetic acid treatment of the lower lip.

After their final peel, patients were followed twice yearly to monitor for signs of recurrence. None of the patients have experienced a recurrence of their actinic cheilitis to date, and the patient who declined a repeat treatment has remained stable at a grade 2. Before and after photos can be seen in Figure 3.

## Discussion

Actinic cheilitis is a premalignant condition affecting the lower lip. It is considered a chronic condition that typically develops in response to longstanding ultraviolet radiation exposure. The reported risk of malignant transformation ranges between 10% and 30%, making early detection and treatment essential.^3^

SCC of the lip is more aggressive than cutaneous SCC, with a high risk for metastasis.^11^, ^12^ The probability of metastasis from cutaneous SCC is 1% compared to 11% for cutaneous SCC of the lower lip.^13^, ^14^ Therefore, it is important to treat actinic cheilitis early to reduce this risk for malignant transformation. Current follow‐up recommendations after actinic cheilitis treatment include twice yearly visits for 2 years followed by annual skin checks after this.^14^

Actinic cheilitis can vary widely in its presentation, ranging from dryness to frank ulceration and crusting. Overt atrophy may be present, and the vermilion border can be poorly defined. There may also be regions of focal hyperkeratosis.^15^

Traditionally, actinic cheilitis has been recognized as a clinical diagnosis. It has been recommended that patients who display exam findings consistent with classic actinic cheilitis should not undergo a biopsy to confirm the diagnosis.^15^ In contrast, persistent and suspicious lesions should undergo biopsy with hematoxylin and eosin staining to rule out malignancy. de Santana Sarmento et al recommended biopsy in the presence of ulcerations/atrophy/nodules, after failure of conservative treatment, or if the area is small and amenable to complete surgical resection.^16^ Basic histopathologic features for actinic cheilitis include hyperkeratosis, solar elastosis, mild to moderate epithelial dysplasia, and perivascular inflammation.^17^ Interestingly, some studies have also suggested a role for biopsy in assessing treatment response. Sotiriou et al report a complete clinical response in 90% of cases with complete histological clearance in only 80%.^18^

The goal of treatment for actinic cheilitis is to reduce the risk of malignant transformation while maintaining lip function and cosmesis. Both medical and surgical options are available. Surgical/ablative techniques include excisional vermilionectomy, electrocautery, laser therapy, or cryotherapy. Although these options achieve high remission rates (92.8%) with a low risk of recurrence (8.4%),^5^ they do come with the associated risk of adverse events including pain, swelling, infection, bleeding, scarring, prolonged healing time, paresthesia, and poor cosmesis. Because of this, surgical vermilionectomy is typically reserved for severe or refractory cases.^19^

Topical treatment is the preferred treatment for patients who have large areas of sun damage in the absence of high‐risk clinical features.^17^ Many treatments, such as imiquimod and 5‐fluorouracil, require repeated applications over a period of weeks.^20^ Unfortunately, this prolonged application period, combined with the expected local side effects, decreases patient compliance.^5^, ^13^ In contrast, TCA can be applied as a single treatment in the majority of patients. Although TCA has been recognized as a topical treatment option for actinic cheilitis, there is currently very little published data regarding its efficacy. This study provides a unique and prospective look at the use of TCA as a treatment regimen.

This study is not without its limitations. As mentioned above, our results rely on clinical judgment for both diagnosis and assessment for remission. Although histopathology would aid in this assessment, we did not wish to subject patients to an additional healing period from a punch biopsy, especially in the posttreatment period when the lip appeared clinically normal. Future studies will aim to incorporate dematoscopic evaluation into the encounter as an additional assessment aid. In addition, we do not currently have a validated grading scale for actinic cheilitis severity. Although we utilized the scale proposed by Poitevin et al,^8^ this scale does not have a category for normal lip, and therefore, the best score we could assign was a grade 1. Future research initiatives should include the development of a validated scale that can be applied to both the pretreatment and posttreatment cohorts. Finally, our small sample size with narrow demographic variability limits external validity. Despite these limitations, our study offers an important look into the efficacy and feasibility of TCA as a treatment option for patients with actinic cheilitis.

## Conclusion

This study suggests that topical TCA treatment for actinic cheilitis is both well tolerated and effective at achieving clinical remission. Patients also reported a significant improvement in the effect of their condition on their quality of life. Although we will continue to monitor for recurrence, all patients in this study who completed the full treatment course are currently disease‐free from a clinical perspective.

## Author Contributions

**Victoria Kuta**, REB, data collection, statistics, manuscript preparation; **S. Mark Taylor**, REB review, TCA peels, patient follow‐up, manuscript preparation.

## Disclosures

### Competing interests

No conflicts to report.

### Funding source

No financial support was provided for this study.

## Supporting information

Appendix A1: Skindex‐16 Survey.

Appendix A2: Proposed grading scale for actinic cheilitis.
