# Supplementary material for: Trichloroacetic Acid as a Topical Treatment for Actinic Cheilitis
Source: OTO Open. 2025 May 23;9(2):e70132. doi: 10.1002/oto2.70132 (PMC12100635; doi:10.1002/oto2.70132)
Supplement: Supplementary file 1 — Appendix A1: Skindex‐16 Survey. [file OTO2-9-e70132-s002.docx]

**THESE QUESTIONS CONCERN THE SKIN CONDITION WHICH HAS BOTHERED YOU THE MOST DURING THE PAST WEEK**

| **During the past week, how often have you been bothered by:** | |  |  |  |  |
| --- | --- | --- | --- | --- | --- |
|  |  | Never Bothered |  | Always Bothered |  |
|  |  | 🡫 | ⚫ | 🡫 |  |
|  |  | ______________________________ | | |  |
| **1.** | Your skin condition **itching** | 🞏_0_ 🞏_1_ 🞏_2_  🞏_3_ 🞏_4_  🞏_5_ 🞏_6_ | | | |
|  |  |  | | | |
| **2.** | Your skin condition **burning** or **stinging** | 🞏_0_ 🞏_1_ 🞏_2_  🞏_3_ 🞏_4_  🞏_5_ 🞏_6_ | | | |
|  |  |  | | | |
| **3.** | Your skin condition **hurting** | 🞏_0_ 🞏_1_ 🞏_2_  🞏_3_ 🞏_4_  🞏_5_ 🞏_6_ | | | |
|  |  |  | | | |
| **4.** | Your skin condition **being irritated** | 🞏_0_ 🞏_1_ 🞏_2_  🞏_3_ 🞏_4_  🞏_5_ 🞏_6_ | | | |
|  |  |  | | | |
| **5.** | The **persistence / reoccurrence** of your skin condition | 🞏_0_ 🞏_1_ 🞏_2_  🞏_3_ 🞏_4_  🞏_5_ 🞏_6_ | | | |
|  |  |  | | | |
| **6.** | **Worry** about your skin condition (For example: that it will spread, get worse, scar, be unpredictable, etc) | 🞏_0_ 🞏_1_ 🞏_2_  🞏_3_ 🞏_4_  🞏_5_ 🞏_6_ | | | |
|  |  |  | | | |
| **7.** | The **appearance** of your skin condition | 🞏_0_ 🞏_1_ 🞏_2_  🞏_3_ 🞏_4_  🞏_5_ 🞏_6_ | | | |
|  |  |  | | | |
| **8.** | **Frustration** about your skin condition | 🞏_0_ 🞏_1_ 🞏_2_  🞏_3_ 🞏_4_  🞏_5_ 🞏_6_ | | | |
|  |  |  | | | |
| **9.** | **Embarrassment** about your skin condition | 🞏_0_ 🞏_1_ 🞏_2_  🞏_3_ 🞏_4_  🞏_5_ 🞏_6_ | | | |
|  |  |  | | | |
| **10.** | **Being annoyed** about your skin condition | 🞏_0_ 🞏_1_ 🞏_2_  🞏_3_ 🞏_4_  🞏_5_ 🞏_6_ | | | |
|  |  |  | | | |
| **11.** | **Feeling depressed** about your skin condition | 🞏_0_ 🞏_1_ 🞏_2_  🞏_3_ 🞏_4_  🞏_5_ 🞏_6_ | | | |
|  |  |  | | | |
| **12.** | The effects of your skin condition on your **interactions with others** (For example: interactions with family, friends, close relationships, etc) | 🞏_0_ 🞏_1_ 🞏_2_  🞏_3_ 🞏_4_  🞏_5_ 🞏_6_ | | | |
|  |  |  | | | |
| **13.** | The effects of your skin condition on your **desire to be with people** | 🞏_0_ 🞏_1_ 🞏_2_  🞏_3_ 🞏_4_  🞏_5_ 🞏_6_ | | | |
|  |  |  | | | |
| **14.** | Your skin condition making it hard to **show affection** | 🞏_0_ 🞏_1_ 🞏_2_  🞏_3_ 🞏_4_  🞏_5_ 🞏_6_ | | | |
|  |  |  | | | |
| **15.** | The effects of your skin condition on your **daily activities** | 🞏_0_ 🞏_1_ 🞏_2_  🞏_3_ 🞏_4_  🞏_5_ 🞏_6_ | | | |
|  |  |  | | | |
| **16.** | Your skin condition making it hard to **work or do what you enjoy** | 🞏_0_ 🞏_1_ 🞏_2_  🞏_3_ 🞏_4_  🞏_5_ 🞏_6_ | | | |
|  |  |  | | | |
