# Supplementary figures and images for: Trichloroacetic Acid as a Topical Treatment for Actinic Cheilitis
Source: OTO Open. 2025 May 23;9(2):e70132. doi: 10.1002/oto2.70132 (PMC12100635; doi:10.1002/oto2.70132)

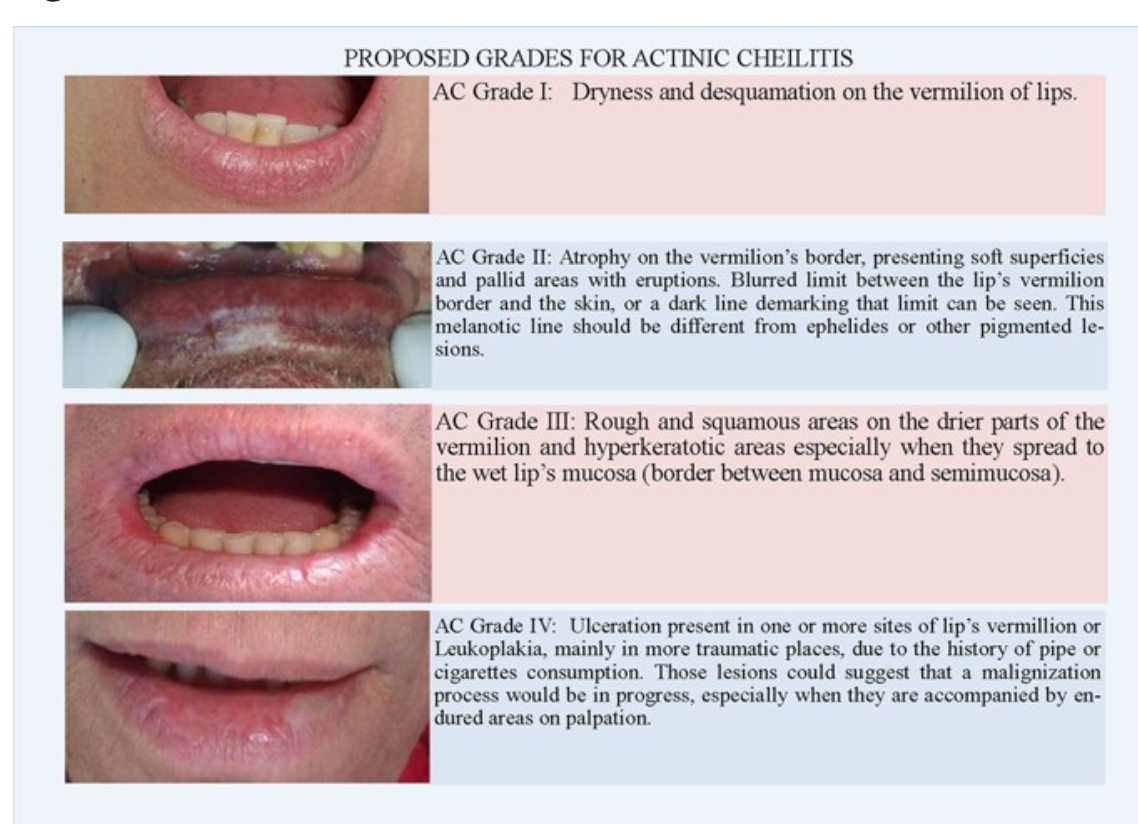

Supplement: Supplementary file 2 — Appendix A2: Proposed grading scale for actinic cheilitis. [file OTO2-9-e70132-s001.docx]
